# Estimation of Behavioral Addiction Prevalence During COVID-19 Pandemic: A Systematic Review and Meta-analysis

**DOI:** 10.1007/s40429-022-00435-6

**Published:** 2022-09-12

**Authors:** Zainab Alimoradi, Aida Lotfi, Chung-Ying Lin, Mark D. Griffiths, Amir H. Pakpour

**Affiliations:** 1 Social Determinants of Health Research Center, Research Institute for Prevention of Non-Communicable Diseases, Qazvin University of Medical Sciences, Qazvin, Iran; 2 Institute of Allied Health Sciences, National Cheng Kung University Hospital, College of Medicine, National Cheng Kung University, Tainan, Taiwan; 3 International Gaming Research Unit, Psychology Department, Nottingham Trent University, Nottingham, UK; 4 Department of Nursing, School of Health and Welfare, Jönköping University, Jönköping, Sweden

**Keywords:** Addictive behavior, COVID-19, Exercise addiction, Food addiction, Internet addiction, Gambling addiction, Gaming addiction, Shopping addiction, Smartphone addiction, Social media addiction

## Abstract

**Purpose of Review:**

The COVID-19 pandemic changed people’s lifestyles and such changed lifestyles included the potential of increasing addictive behaviors. The present systematic review and meta-analysis aimed to estimate the prevalence of different behavioral addictions (i.e., internet addiction, smartphone addiction, gaming addiction, social media addiction, food addiction, exercise addiction, gambling addiction, and shopping addiction) both overall and separately.

**Recent Findings:**

Four databases (*PubMed*, *Scopus, ISI Web of Knowledge*, and *ProQuest*) were searched. Peer-reviewed papers published in English between December 2019 and July 2022 were reviewed and analyzed. Search terms were selected using PECO-S criteria: population (no limitation in participants’ characteristics), exposure (COVID-19 pandemic), comparison (healthy populations), outcome (frequency or prevalence of behavioral addiction), and study design (observational study). A total of 94 studies with 237,657 participants from 40 different countries (mean age 25.02 years; 57.41% females). The overall prevalence of behavioral addiction irrespective of addiction type (after correcting for publication bias) was 11.1% (95% *CI*: 5.4 to 16.8%). The prevalence rates for each separate behavioral addiction (after correcting for publication bias) were 10.6% for internet addiction, 30.7% for smartphone addiction, 5.3% for gaming addiction, 15.1% for social media addiction, 21% for food addiction, 9.4% for sex addiction, 7% for exercise addiction, 7.2% for gambling addiction, and 7.2% for shopping addiction. In the lockdown periods, prevalence of food addiction, gaming addiction, and social media addiction was higher compared to non-lockdown periods. Smartphone and social media addiction was associated with methodological quality of studies (i.e., the higher the risk of boas, the higher the prevalence rate). Other associated factors of social media addiction were the percentage of female participants, mean age of participants, percentage of individuals using the internet in country, and developing status of country. The percentage of individuals in the population using the internet was associated with all the prevalence of behavioral addiction overall and the prevalence of sex addiction and gambling addiction. Gaming addiction prevalence was associated with data collection method (online vs. other methods) that is gaming addiction prevalence was much lower using online methods to collect the data.

**Summary:**

Behavioral addictions appeared to be potential health issues during the COVID-19 pandemic. Healthcare providers and government authorities should foster some campaigns that assist people in coping with stress during COVID-19 pandemics to prevent them from developing behavioral addictions during COVID-19 and subsequent pandemics.

**Supplementary Information:**

The online version contains supplementary material available at 10.1007/s40429-022-00435-6.

## Introduction

Numerous research studies have been conducted since the advent of the COVID-19 pandemic to identify the various effects and impacts of this new disease [1]. The COVID-19 pandemic has had a rapid and varied impact on many aspects of the personal, family, social, occupational, and economic lives of many people [2–6]. Social, financial, health, job, and other epidemic-related stressors may motivate individuals to engage in potentially addictive behaviors, including internet use [7], gambling [8], online shopping [9•], online gaming [10], eating [11], exercise [12], and even work [13]. Such addictive behaviors could be viewed as a type of coping strategies for individuals to shift their attention from fear, anxiety, and/or worry about COVID-19 to other activities. Moreover, given that some strong and unprecedented policies in COVID-19 infection control have been implemented (e.g., lockdowns, quarantine, and closures of educational and occupational buildings), individuals were forced to live in a lifestyle they had never experienced before [14–16]. Therefore, these potentially addictive behaviors may also have helped individuals to cope with the new lifestyles they experienced during the COVID-19 pandemic.

As proposed in the Interaction of Person-Affect-Cognition-Execution (I-PACE) model [17], individuals engage in problematic internet use behaviors (potentially a type of addiction) because they can use activities on the internet to cope with their psychological distress. Subsequently, individuals can get themselves into a vicious cycle where they engage in internet use to cope with psychological distress, but then being on the internet all the time causes conflicts in their lives, and the only way to deal with the conflicts is to spend more time on the internet. For a minority of individuals, this could develop into an internet addiction. The same mechanisms could also explain why other potentially addictive behaviors may have been used by individuals during the COVID-19 pandemic (i.e., they use these behaviors to cope with the high levels of psychological distress caused by COVID-19).

The COVID-19 pandemic has provided an unprecedented opportunity for researchers worldwide to study the impact of stressful life events on individuals’ psychological responses and addictive behaviors [18]. During the COVID-19 pandemic, various measures were taken to control the disease and reduce mortality, including travel restrictions and quarantine, as well as the closure of schools, public spaces, and workplaces [19]. During this period, young people were forced to spend large amounts of daily time in front of screens such as tablets, smartphones, desktops, and televisions just so that they could continue to be educated [20, 21].

Spending time online among young people has traditionally been leisure-related. According to a German study, children between the ages of 10 and 17 years played significantly more video games during quarantine vs. pre-pandemic times [22]. Moreover, other studies have reported the increased time spent on internet-related activities (such as gaming, social media use, and smartphone use) during the pandemic compared to time spent online before it [23–26]. This has been of concern in relation to the use of technology and subsequent addictive behaviors [27–29]. Therefore, it is important to understand the severity of such addictive behaviors during the COVID-19 pandemic.

Even before the COVID-19 pandemic, evidence has been cumulated to indicate the important issues of behavioral addictions. More specifically, evidence before the pandemic shows that internet addiction had a prevalence rate of 6.0% (95% *CI* 5.1–6.9) in a meta-analysis [30]; gaming addiction had a prevalence rate of about 6.0% in a meta-analysis [31]; gambling addiction had a prevalence rate between 2.7 and 4.2% in a meta-analysis [32]; shopping addiction had a prevalence rate of 4.9% (95% *CI* 3.4–6.9) [33]; food addiction had a prevalence rate of 16.2% (95% *CI* 13.6–19.3) in a meta-analysis [34]; exercise addiction had a prevalence rate about 3% in a narrative review [35]; social media addiction had a prevalence rate between 1.6 and 34.0% in a narrative review [36]; and smartphone addiction had a prevalence rate of 23.3% (95% *CI* 14.0–31.2) in a meta-analysis [37].

Apart from the rates of prevalence, empirical evidence and discussions prior to the COVID-19 pandemic show that examining these behavioral addictions is important. For example, the 5th edition of the Diagnostic and Statistical Manual of Mental Disorders (DSM-5) has begun to acknowledge the importance of behavioral addictions [38, 39], and the social impacts of behavioral addictions have led to growing interest that need further evidence investigating its pathophysiological mechanism [40–42], comorbidity between psychiatric disorders and behavioral addictions [43, 44], and the potential treatments of behavioral addictions [45, 46]. Therefore, the evidence and discussions prior to COVID-19 pandemic additionally support the importance of investigating behavioral addictions during the pandemic.

To the best of the present authors’ knowledge, there has been no previous systematic review and meta-analysis to estimate the overall prevalence of behavioral addictions during the COVID-19 pandemic (e.g., internet addiction, gambling addiction, shopping addiction, food addiction, exercise addiction, social media addiction, and smartphone addiction). The issues of these different types of behavioral addictions have been identified with several statements claiming the importance to take care of the time spent on these behaviors during the COVID-19 pandemic [47–49]. However, without empirical evidence showing how severe these behavioral addictions were during the COVID-19 pandemic, government authorities might not take such statements seriously. Therefore, the present study used a rigorous and robust method to search the literature reporting prevalence/frequency for different types of behavioral addiction during the COVID-19 pandemic. Moreover, in the present systematic review and meta-analysis, the term “addiction” was used. Although many studies used other terms (e.g., problematic use, dependence, and disorder) to indicate each behavior problem, “addiction” was used with the consideration of easy-understanding for all different behaviors assessed in the present study. That is, “behavioral addictions” itself is a well-recognized term and can be easily understood by all the experts in the field, although not everyone accepts using this term.

## Methods

### Design and Registration

The present systematic review and meta-analysis were carried out based on the Preferred Reporting Items for Systematic Reviews and Meta-Analyses (PRISMA) guidelines [50]. The protocol of the present study was prospectively registered within international prospective register of systematic reviews PROSPERO (Decree code: CRD42022330898) [51].

### Search Strategy

Four major academic databases were systematically searched using the publication period between December 2019 and May 2022 (i.e., *PubMed, Scopus, ISI Web of Knowledge,* and *ProQuest*). Search syntax was developed using main search terms from PubMed Medical Subject Headings (MeSH). Main search terms were selected based on PECO-S search strategy (i.e., population, exposure, comparison, outcome, and study design) [52]. In the present study, two main components of exposure (COVID-19 pandemic) and outcome (each type of behavioral addiction) were selected. The main search terms were (internet OR “social media” OR smartphone OR “mobile phone” OR “cell phone” OR gaming OR “video gam*” OR “social network*” OR Twitter OR Instagram OR “YouTube” OR “Facebook” OR “WhatsApp” OR “TikTok” OR “WeChat” OR “SnapChat” OR “QQ” OR “Tinder” OR gambl* OR betting OR “electronic gaming machines” OR lotto OR casino OR poker OR bingo OR blackjack OR lottery OR “slot machine*” OR exercis* OR “physical activity” OR pornography OR sex* OR food OR “binge eating” OR mukbang OR shopping OR buying OR technolog*) AND (addict* OR problem* OR depend* OR disorder* OR obsess* OR excess* OR compuls* OR impuls* OR excess*) AND (“SARS-CoV-2” OR “coronavirus” OR “COVID-19” OR “2019-nCoV” OR “coronavirus disease-2019” OR covid OR coronavirus OR “2019-ncov” OR “sars-cov-2” OR “cov-19”). Search strategy was customized for each database according to its advanced search attributes (provided in Supplementary Materials 1). To increase comprehensiveness of search, reference lists of included studies and published systematic reviews as well as the first ten pages of *Google Scholar* for each type of behavioral addiction were hand searched.

### Eligibility Criteria

The eligibility criteria were constructed based on PECO-S components:

- Population: Individuals with any age or gender group (in other words, no limitation regarding participants’ characteristics).
- Exposure: COVID-19 pandemic.
- Comparison: Healthy population.
- Outcome: Frequency or prevalence of any type of behavioral addiction. However, behavioral addictions should be assessed using valid and reliable measures.
- Study design: Observational studies reporting data on frequency or prevalence of any type of behavioral addiction among participants.

Eligible papers were those published between December 2019 and July 2022 using English language and had been published in peer-reviewed papers.

### Outcomes

#### Primary Outcome

Estimates of behavioral addiction prevalence during the COVID-19 pandemic were the primary outcome. Behavioral addiction could be considered as a specific condition that involves mental and behavioral disorders [53]. Therefore, behavioral addiction is defined as a set of coercive behaviors in which a person feels compelled to do something, although the individual knows that engaging in such behaviors may harm them and causes clinical impairment of individuals’ day-to-day activities [54]. There are different types of behavioral addiction, such as internet use, gambling, gaming, shopping, binge eating/food eating, sex, smartphone use, exercise, and work [55]. The primary outcome combined all the types of behavioral addiction for prevalence estimation.

#### Secondary Outcomes

i. Prevalence of each type of behavioral addictions.
ii. Assessing the possible sources of heterogeneity.
iii. Investigating the predictor variables of behavioral addiction prevalence.

### Study Screening and Selection

Two independent reviewers screened the title and abstract of retrieved papers based on the eligibility criteria. The full texts of potentially relevant studies were further examined based on the aforementioned criteria. In this process, relevant studies were selected for further analysis.

### Quality Assessment

The methodological quality of included studies was assessed using the Newcastle Ottawa Scale (NOS). Three main methodological characteristics of selection, comparability, and outcome assessment are examined with the NOS checklist. There are three versions of the checklist for evaluating cross-sectional studies (7 items), case–control (8 items), and cohort (8 items). Despite a slight difference in the number and content of these items, each item is rated with one point (except for comparability, which can have two points) for a maximum possible score of 9. Studies with less than 5 points are classified as having a high risk of bias [56]. No studies were excluded based on the quality rating. However, the impact of quality on pooled effect size was assessed via meta-regression.

### Data Extraction

A pre-designed Excel spreadsheet was prepared to extract data. The following items were extracted: first author’s name, publication and data collection dates, study design, country (or countries) where data were collected, number of participants, mean age, scales used to assess behavioral addiction, data collection method, countries’ developmental and income status based on world bank reports, and numerical results regarding the frequency of both overall behavioral addiction and types of specified behavioral addiction. It should also be noted that study selection, quality assessment, and data extraction were processes performed independently by two reviewers. Disagreements were resolved through discussion.

### Data Synthesis

Evidence from included studies was quantitatively synthesized using STATA software version 14. As included studies were from different populations, meta-analysis using a random effects model was conducted to account for both within-study and between-study variances [57]. Severity of heterogeneity was estimated using the *I*^2^ index [58]. Prevalence of behavioral addiction and its 95% confidence intervals (*CI*) were the selected key measure for the present study. To investigate predictor variables for behavioral addiction, meta-regression was conducted. Funnel plot and Begg’s Test were used to assess publication bias [59]. Meta-trim with the fill and trim method was used to correct probable publication bias [60]. The Jackknife method was used for sensitivity analysis and probable single study effect on pooled effect size [61]. Uni-variable and multivariable meta-regression was used to assess moderators of behavioral addiction prevalence. When values of adjusted *R*^2^ were considerable for examined variable in uni-variable regression, they were entered in multivariable meta-regression models.

## Results

### Study Screening and Selection Process

**Fig. 1:**
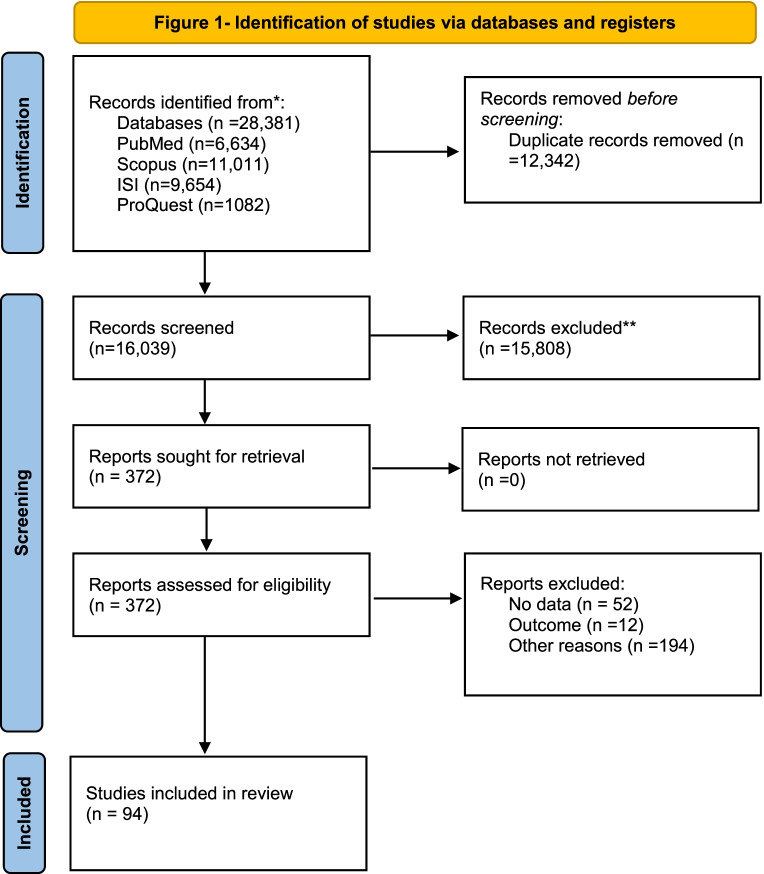
Identification of studies via databases and registers

The initial search in four academic databases resulted in 28,381 papers: *PubMed* (*n* = 6,634), *Scopus* (*n* = 11,011), *ISI Web of Knowledge* (*n* = 9654), and *ProQuest* (*n* = 1082). After removing duplicates (*n* = 12,342), the remaining papers were screened based on their title and abstract. Finally, 372 papers appeared to be potentially eligible and their full-texts were reviewed. In this process, 94 studies met the eligibility criteria and were pooled in the meta-analysis. Figure 1 shows the search process based on the PRISMA flowchart.

### Study Description

A total of 94 studies with 237,657 participants from 40 different countries (Argentina, Australia, Bangladesh, Brazil, Chile, China, Costa Rica, Croatia, Dominican Republic, Ecuador, Egypt, Guyana, Honduras, Hungary, India, Indonesia, Iran, Italy, Japan, Jordan, Kuwait, Lebanon, Lithuania, Malaysia, Mexico, Pakistan, Peru, Poland, Russian, Saudi Arabia, Spain, Sudan, Sweden, Switzerland, Taiwan, Turkey, UK, USA, Uruguay, and Vietnam) were included. A total of 27 studies gathered data during the national lockdown period in their respective countries. The smallest sample size was 42 (from the USA), and the largest sample size was 51,246 (from Japan). The mean age of participants was 25.02 years with age range between 5 and 82 years. Almost all studies used a cross-sectional design. One study was a longitudinal study with three waves in COVID-19 pandemic; data regarding each wave was extracted as a separate study. Three papers reported the results from multi-countries and 27 studies were population-based. Most studies (49 out of 94) were conducted in developed countries. All studies had participants from both gender groups with 57.41% female. The main behavioral addictions studied were internet use (39 studies), gaming (19 studies), gambling (18 studies), smartphone use (13 studies), social media use (10 studies), food addiction (five studies), exercise (four studies), sex addiction (four studies), and shopping addiction (two studies). Fourteen studies reported more than one type of behavioral addiction. No study was retrieved regarding the prevalence of work addiction. Table 1 provides the summary characteristics of all included studies.

**Table 1:** Summarized characteristics of included studies

Author	Publication year/data collection time	Country Development status Income level Individuals using the Internet (% of population)	Data collection method	Lock down	Participant group	Mean age	Sample size/female %	Measures	Type of behavioral addiction	NOS total/category	Population-based study
**i. Internet addiction**											
Truzoli [62]	2021/	Italy Developed High income 74.39	Online	Yes	Students	19.3	191/73.3	IAT	Internet	5/high risk of bias	No
Tahir [63•]	2021/2020	Multi Country Developing Lower intermediate income	Online	No	General population	NR	2749/64	IAT	Internet	6/low risk of bias	Yes
Ozturk [64]	2021/2020	Turkey Developed Upper intermediate income 73.98	Online	No	Students	NR	1572/63.9	Parent–child IAT	Internet	7/low risk of bias	No
Aközlü [65]	2021/2020	Turkey Developed Upper intermediate income 73.98	Questionnaire	No	Students	8.52	154	Parent–child IAT	Internet	6/low risk of bias	No
Kamaşak [66]	2022/2021	Turkey Developed Upper intermediate income 73.98	Online	No	Children	13	4892/51.6	Parent–child IAT	Internet	7/low risk of bias	No
Perez-Siguas [67]	2021/	USA Developed High income 88.5	Online	No	Voluntarily participate	NR	113/71.7	IAT	Internet	3/high risk of bias	No
Gansner [68]	2022/2020	USA Developed High income 88.5	Online	No	Adolescents with psychiatric disorders	16.95	42/76.2	PRIUSS	Internet	5/high risk of bias	No
Lakkunarajah [69]	2022/2021	USA Developed High income 88.5	Questionnaire	No	Adolescents with psychiatric disorders	16	447/96	PRIUSS	Internet	6/low risk of bias	No
Siste [70]	2021/2020	Indonesia Developing Lower intermediate income 47.69	Online	Yes	Students	17.38	2932/78.7	IAT	Internet	5/high risk of bias	No
Siste [71]	2020/2020	Indonesia Developing Lower intermediate income 47.69	Online	No	Adults	31.84	4734/44.8	IAT	Internet	5/high risk of bias	No
Jiang [72]	2022/	China Developing Upper intermediate income 54.3	Questionnaire	No	University students	20.49	2688	IAT	Internet	8/low risk of bias	No
Li [73•]	2021/2020	China Developing Upper intermediate income 54.3	Online	No	General population	33.63	20,472/56.5	IAT	Internet	6/low risk of bias	Yes
Zhu [74]	2020/2020	China Developing Upper intermediate income 54.3	Online	No	University students	20.56	7562/54.4	YDQ	Internet	6/low risk of bias	No
Li [75]	2021/2020	China Developing Upper intermediate income 54.3	Face-to-face interview	No	Adolescents with psychiatric disorders	14.73	1454/61.2	IAT	Internet	6/low risk of bias	No
Wu [76]	2021/2020	China Developing Upper intermediate income 54.3	Online	No	Students	14.9	625/50.7	IAT	Internet	6/low risk of bias	No
Liang [77]	2022/2020	China Developing Upper intermediate income 54.3	Online	No	Youth	22.28	552/ 63.4	IAT	Internet	7/low risk of bias	No
Dong [18]	2020/2020	China Developing Upper intermediate income 54.3	Online	No	Students	12.34	2050/48.44	IAT	Internet	6/low risk of bias	No
Cai [78]	2021/2020	China Developing Upper intermediate income 54.3	Online	No	University students	19.7	1070/75.2	IAT	Internet	6/low risk of bias	No
Sun [79]	2020/2020	China Developing Upper intermediate income 54.3	Online	No	General population	28.23	6416/53	IAT	Internet	3/high risk of bias	Yes
Zhao [80]	2021/2020	China Developing Upper intermediate income 54.3	Online	No	University students	20	11,254/64	IAT	Internet	6/low risk of bias	No
Liu [81]	2022/2020	China Developing Upper intermediate income 54.3	Online	Yes	Students	13.8	4852/51.5	IAT	Internet	6/low risk of bias	No
Xia [82]	2021/2020	China Developing Upper intermediate income 54.3	Online	Yes	University students	19.69	494/71.5	IAT	Internet	6/low risk of bias	No
Xie [83]	2021/2020	China Developing Upper intermediate income 54.3	Online	No	University students	21.27	8879/54.4	YDQ	Internet	6/low risk of bias	No
Shehata [84]	2021/2020	Egypt Developing Lower intermediate income 57.28	Questionnaire	No	University students	NR	746/67.16	IAT	Internet	7/low risk of bias	No
AlSumait [85]	2021/	Middle East Developed Upper intermediate income 65.14	Online	Yes	voluntarily participate	NR	613/68.9	IAT	Internet	4/high risk of bias	No
Jahan [86]	2021/2020	Bangladesh Developing Lower intermediate income 18.02	Online	No	Students	NR	601/42.8	IAT	Internet	7/low risk of bias	No
Nakayama [87]	2021/2020	Japan Developed High income 91.28	Questionnaire	No	Students	NR	802/48.9	YDQ	Internet	6/low risk of bias	No
Lin [88]	2020/ = 2020	Taiwan Developed High income 91	Questionnaire	No	Students	14.72	1042/48.36	IAT	Internet	6/low risk of bias	No
Prakash [89]	2020/2020	India Developing Lower intermediate income 32.00	Online	Yes	General population	27.69	350/34.6	IAT	Internet	7/low risk of bias	Yes
Meitei [90]	2021/2020	India Developing Lower intermediate income 32.00	Online	Yes	General population	NR	585	IAT	Internet	7/low risk of bias	Yes
Gecaite-Stonciene [91]	2021/2020	Lithuania Developed High income 81.58	Online	No	University students	22	619/92.9	PRIUSS	Internet	6/low risk of bias	No
Vejmelka [92]	2021/2020	Croatia Developing High income 79.08	Online	No	Students	14.97	494/57.3	IAT	Internet	7/low risk of bias	No
Volpe [93]	2022/2022	Italy Developed High income 74.39	Online	Yes	Adults	32.5	1385/62.5	IAT; IGDS; BSMAS	Internet	6/low risk of bias	No
Ismail [94]	2021/2020	Malaysia Developed Upper intermediate income 84.21	Online	No	University students	NR	237/69.6	IAT; IGDS	Internet	6/low risk of bias	No
Oka [95]	2021/2020	Japan Developed High income 91.28	Online	No	Adults	46.6	51,246/50.1	CIUS; IGDS	Internet	6/low risk of bias	No
Ballarotto [96]	2021/2020	Italy Developed High income 74.39	Online	No	Adults	22.96	400/70	IAT; BSMAS	Internet	5 /high risk of bias	No
Duan [97]	2020/2020	China Developing Upper intermediate income 54.3	Online	No	Students	NR	3183/49.85	IAT; SAS-SF	Internet	6/low risk of bias	No
**ii. Gaming addiction**											
Saritepeci [98]	2022/2021	Turkey Developed Upper intermediate income 73.98	Online	No	University students	21.35	588/69.6	IAT	Gaming	5/high risk of bias	No
Çakıroğlu [99]	2021/2020	Turkey Developed Upper intermediate income 73.98	Online	No	Students	13.7	410/56.3	IGDS	Gaming	6/low risk of bias	No
Nugraha [100]	2021/2020	Indonesia Developing Lower intermediate income 47.69	Online	No	Students	NR	136/36.76	GAS-A	Gaming	4/high risk of bias	No
Chang [101]	2022/2020	China Developing Upper intermediate income 54.3	Online	No	Students	15.16	1305/41.5	IGDS	Gaming	6/low risk of bias	No
Zhu [10]	2021/2020	China Developing Upper intermediate income 54.3	Questionnaire	Yes	Students	12.6	2863/52.7	CGAS-SF	Gaming	7/low risk of bias	No
Wu [102]	2022/2020	China Developing Upper intermediate income 54.3	Online	No	General population	27	5268/47.4	IGDS	Gaming	6/low risk of bias	Yes
Teng [103]	2021/2020	China Developing Upper intermediate income 54.3	Online	Yes	Students	NR	1778/49.3	IGDS	Gaming	7/low risk of bias	No
Galán [104]	2021/2021	Spain Developed High income 90.72	online	No	University students	23.7	310/69.9	GAS-A	Gaming	5/high risk of bias	No
Duong [105]	2021/2020	Vietnam Developing Lower intermediate income 68.7	Questionnaire	No	Students	14.5	2084/50.2	IGDS	Gaming	7/low risk of bias	No
Zaman [106]	2022/2020	Pakistan Developing Lower intermediate income 17.07	Online	Yes	General population	25	618/32.52	GAS	Gaming	7/low risk of bias	Yes
Fazeli [107]	2020/2020	Iran Developing Lower intermediate income 70	Online	Yes	Students	15.51	1512/44.6	IGDS	Gaming	6/low risk of bias	No
Volpe [93]	2022/2022	Italy Developed High income 74.39	Online	Yes	Adults	32.5	1385/62.5	IAT; IGDS; BSMAS	Gaming	6/low risk of bias	No
Ismail [94]	2021/2020	Malaysia Developed Upper intermediate income 84.21	Online	No	University students	NR	237/69.6	IAT; IGDS	Gaming	6/low risk of bias	No
She [108]	2022/2020	China Developing Upper intermediate income 54.3	Questionnaire	No	Students	13.6	3136/51.9	PBS	Gaming	6/low risk of bias	No
Forster [109]	2021/2020	USA Developed High income 88.5	Email	No	University students	NR	1027/78.32	IAT; SAS-SF	Gaming	6/low risk of bias	No
Oka [95]	2021/2020	Japan Developed High income 91.28	Online	No	Adults	46.6	51,246/50.1	CIUS; IGDS	Gaming	6/low risk of bias	No
Koós Wave 1 [110•]	2022/2020	Hungary Developed High income 80.37	Online	Yes	General population	41.96	1747/49.5	PGSI; IGDS; BSMAS; CSBDS	Gaming	6/low risk of bias	Yes
Koós Wave 2 [110•]	2022/2020	Hungary Developed High income 80.37	Online	Yes	General population	41.96	656/49.5	PGSI; IGDS; BSMAS; CSBDS	Gaming	6/low risk of bias	Yes
Koós Wave 3 [110•]	2022/2020	Hungary Developed High income 80.37	Online	Yes	General population	41.96	411/49.5	PGSI; IGDS; BSMAS; CSBDS	Gaming	6/low risk of bias	Yes
Claesdotter-Knutsson [111]	2022/2021	Sweden Developed High income 94.49	Online	No	General population	NR	932/48.5	PGSI; GAS-A	Gaming	6/low risk of bias	Yes
Chen [23]	2021/2020	China Developing Upper intermediate income 54.3	Online	No	Students	11.29	504/50	SABAS; BSMAS; IGDS	Gaming	6/low risk of bias	No
**iii. Gambling addiction**											
Amerio [112]	2021/2021	Italy Developed High income 74.39	Online	Yes	General population	NR	6003/50.66	Pacifici et al. 2019	Gambling	5/high risk of bias	Yes
Salerno [113]	2021/2020	USA Developed High income 88.5	Online	No	General population	33.65	254/55.9	PG adaptation of Yale-Brown OCS	Gambling	6/low risk of bias	Yes
Xuereb [114]	2021/2020	USA Developed High income 88.5	Online	Yes	Gamblers in past 12 month	37.93	424/36.1	PGSI	Gambling	6/low risk of bias	No
Håkansson [115]	2020/2020	Sweden Developed High income 94.49	Online	Yes	Gamblers in past 12 month	NR	997/25	PGSI	Gambling	5/high risk of bias	No
Månsson [116]	2021/2020	Sweden Developed High income 94.49	Online	Yes	Gamblers in past 12 month	39.8	325/35.2	PGSI	Gambling	5/high risk of bias	No
Claesdotter-Knutsson [117]	2021/2021	Sweden Developed High income 94.49	Online	Yes	General population	NR	1064/44	PGSI	Gambling	6/low risk of bias	Yes
Håkansson [118]	2021/2020	Sweden Developed High income 94.49	Online	No	General population	NR	2029/52	PGSI	Gambling	6/low risk of bias	Yes
Håkansson [119]	2020/2020	Sweden Developed High income 94.49	Email	No	Elite athletes	NR	327/36.09	PGSI	Gambling	5/high risk of bias	No
Håkansson [120]	2020/2020	Sweden Developed High income 94.49	Online	No	General population	NR	2016/49	PGSI	Gambling	6/low risk of bias	Yes
Wardle [121]	2021/2020	UK Developed High income 92.52	Online	Yes	people who bet regularly (at least monthly) on sports before COVID-19	NR	3866/20.23	PGSI	Gambling	5/high risk of bias	No
Sharman [122]	2021/2020	UK Developed High income 92.52	Online	No	General population	33.19	1028/72.1	BPGS	Gambling	6/low risk of bias	Yes
Lischer [123]	2021/2020	Switzerland Developed High income 93.15	Email	No	Gamblers in past 12 month	33.5	110/22.7	SOGS	Gambling	5/high risk of bias	No
Gainsbury [124]	2021/2020	Australia Developed High income 86.55	Online	No	Gamblers in past 12 month	43.8	764/14.4	PGSI	Gambling	6/low risk of bias	No
Zamboni [9•]	2021/2020	Italy Developed High income 74.39	Online	No	General population	43.25	1196/64.6	One item asking about of control of the behavior	Gambling	1/high risk of bias	Yes
Koós Wave 1 [110•]	2022/2020	Hungary Developed High income 80.37	Online	Yes	General population	41.96	1747/49.5	PGSI; IGDS; BSMAS; CSBDS	Gambling	6/low risk of bias	Yes
Koós Wave 2 [110•]	2022/2020	Hungary Developed High income 80.37	Online	Yes	General population	41.96	656/49.5	PGSI; IGDS; BSMAS; CSBDS	Gambling	6/low risk of bias	Yes
Koós Wave 3 [110•]	2022/2020	Hungary Developed High income 80.37	Online	Yes	General population	41.96	411/49.5	PGSI; IGDS; BSMAS; CSBDS	Gambling	6/low risk of bias	Yes
Claesdotter-Knutsson [111]	2022/2021	Sweden Developed High income 94.49	Online	No	General population	NR	932/48.5	PGSI; GAS-A	Gambling	6/low risk of bias	Yes
**iv. Smartphone addiction**											
Serra [125]	2021/2020	Italy Developed High income 74.39	Online	No	Students	4.84	184/71.7	SAS-SF	Smartphone	6/low risk of bias	No
Indrakusuma [126]	2021/2020	Indonesia Developing Lower intermediate income 47.69	Online	No	University students	NR	364/79.4	SAS-SF	Smartphone	6/low risk of bias	No
Zhang [127]	2021/2020	China Developing Upper intermediate income 54.3	Online	No	University students	26.01	1016/65.16	SAS-SF	Smartphone	6/low risk of bias	No
Hu [128]	2021/2020	China Developing Upper intermediate income 54.3	Online	No	Students	16.53	2090/62.4	MPAI	Smartphone	6/low risk of bias	No
Zhao [129]	2022/2021	China Developing Upper intermediate income 54.3	Online	No	University students		500/66.4	SAS-SF	Smartphone	6/low risk of bias	No
Elhai [130]	2020/2020	China Developing Upper intermediate income 54.3	Online	No	Adults	41.32	908/82.82	SAS-SF	Smartphone	6/low risk of bias	No
Duan [131]	2021/2020	China Developing Upper intermediate income 54.3	Online	No	Students	NR	3615/50.2	SAS-SF	Smartphone	6/low risk of bias	No
Saadeh [132]	2021/2020	Jordan Developing Upper intermediate income 66.79	Online	No	University students	19.79	6157/71.3	SAS-SF	Smartphone	6/low risk of bias	No
Hosen [133]	2021/2020	Bangladesh Developing Lower intermediate income 18.02	Online	No	Students	NR	601/42.8	SAS-SF	Smartphone	5/high risk of bias	No
Sfeir [134]	2021/2020	Lebanon Developing Upper intermediate income 78.18	Online	Yes	Adults	22.25	461/70.9	SAS-SF	Smartphone	7/low risk of bias	No
Perez-Siguas [135]	2020/2020	Peru Developing Upper intermediate income 59.95	Online	No	Students	NR	163/71.17	MPPUS	Smartphone	6/low risk of bias	No
Forster [109]	2021/2020	US Developed High income 88.5	Email	No	University students	NR	1027/78.32	IAT; SAS-SF	Smartphone	6/low risk of bias	No
Duan [97]	2020/2020	China Developing Upper intermediate income 54.3	Online	No	Students	NR	3183/49.85	IAT; SAS-SF	Smartphone	6/low risk of bias	No
Chen [23]	2021/2020	China Developing Upper intermediate income 54.3	Online	No	Students	11.29	504/50	SABAS; BSMAS; IGDS	Smartphone	6/low risk of bias	No
**v. Social media addiction**											
Duran [136]	2022/2021	Turkey Developed Upper intermediate income 73.98	Online	No	Adults	NR	405	BSMAS	Social media	7/low risk of bias	Yes
Luo [137]	2021/2020	China Developing Upper intermediate income 54.3	Online	No	General population	33.38	10,963/57.22	BSMAS	Social media	6/low risk of bias	Yes
Lin [138]	2020/2020	Iran Developing Lower intermediate income 70	Online	No	Students	26.24	1078/58.3	BSMAS	Social media	5/high risk of bias	No
Volpe [93]	2022/2022	Italy Developed High income 74.39	Online	Yes	Adults	32.5	1385/62.5	IAT; IGDS; BSMAS	Social media	6/low risk of bias	No
Panno [139•]	2020/2020	Italy Developed High income 74.39	Online	Yes	General population	28.49	1519/76	BSMAS; YFAS	Social media	6/low risk of bias	Yes
She [108]	2022/2020	China Developing Upper intermediate income 54.3	Questionnaire	No	Students	13.6	3136/51.9	PBS	Social media	6/low risk of bias	No
Ballarotto [96]	2021/2020	Italy Developed High income 74.39	Online	No	Adults	22.96	400/70	IAT; BSMAS	Social media	5/high risk of bias	No
Koós Wave 1 [110•]	2022/2020	Hungary Developed High income 80.37	Online	Yes	General population	41.96	1747/49.5	PGSI; IGDS; BSMAS; CSBDS	Social media	6/low risk of bias	Yes
Koós Wave 2 [110•]	2022/2020	Hungary Developed High income 80.37	Online	Yes	General population	41.96	656/49.5	PGSI; IGDS; BSMAS; CSBDS	Social media	6/low risk of bias	Yes
Koós Wave 3 [110•]	2022/2020	Hungary Developed High income 80.37	Online	Yes	General population	41.96	411/49.5	PGSI; IGDS; BSMAS; CSBDS	Social media	6/low risk of bias	Yes
Chen [23]	2021/2020	China Developing Upper intermediate income 54.3	Online	No	Students	11.29	504/50	SABAS; BSMAS; IGDS	Social media	6/low risk of bias	No
**vi. Food addiction**											
Borisenkov [140]	2020/2020	Russia Developing Upper intermediate income 82.64	Online	No	University students	21.8	949/78.3	YFAS	Food	6/low risk of bias	No
da Silva Júnior AE [141]	2021/2021	Brazil Developed Upper intermediate income 70.43	Online	No	University students	24.1	5368/74.3	YFAS	Food	7/low risk of bias	No
Schulte [142]	2022/2021	USA Developed High income 88.5	Online	No	General population	42.36	288/54.5	YFAS	Food	5/high risk of bias	Yes
Zielinska [143]	2021/2021	Poland Developed High income 84.52	Online	No	General population	33.18	1022/93.7	YFAS	Food	7/low risk of bias	Yes
Panno [139•]	2020/2020	Italy Developed High income 74.39	Online	Yes	General population	28.49	1519/76	BSMAS; YFAS	Food	6/low risk of bias	Yes
**vii. Sex addiction**											
Caponnetto [144]	2022/2021	Italy Developed High income 74.39	Online	Yes	General population	23.1	1401/52	SAST	Sex addiction	6/low risk of bias	Yes
Zamboni [9•]	2021/2020	Italy Developed High income 74.39	Online	No	General population	43.25	1196/64.6	One item asking about of control of the behavior	Sex addiction	1/high risk of bias	Yes
Koós Wave 1 [110•]	2022/2020	Hungary Developed High income 80.37	Online	Yes	General population	41.96	1747/49.5	PGSI; IGDS; BSMAS; CSBDS	Sex addiction	6/low risk of bias	Yes
Koós Wave 2 [110•]	2022/2020	Hungary Developed High income 80.37	Online	Yes	General population	41.96	656/49.5	PGSI; IGDS; BSMAS; CSBDS	Sex addiction	6/low risk of bias	Yes
Koós Wave 3 [110•]	2022/2020	Hungary Developed High income 80.37	Online	Yes	General population	41.96	411/49.5	PGSI; IGDS; BSMAS; CSBDS	Sex addiction	6/low risk of bias	Yes
**viii. Exercise addiction**											
Ceci [145]	2022/2020	Italy Developed High income 74.39	Online	Yes	General population	31.54	782/66	EAI	Exercise	6/low risk of bias	Yes
Cataldo [146]	2022/2020	Multi country Developed High income	Online	Yes	Adults	37.75	729/72.3	EAI	Exercise	5/high risk of bias	No
de la Vega [147•]	2020/2020	Multi country Developed High income	Online	No	General population	32.88	1079/48	EAI	Exercise	5/high risk of bias	Yes
Berengüí [148]	2021/2020	Spain Developed High income 90.72	Questionnaire	Yes	General population	35.1	1019/47.8	EAI	Exercise	4/high risk of bias	Yes
**ix. Shopping addiction**											
Duong [105]	2021/	Vietnam Developing Lower intermediate income 68.7	Online	No	University students	NR	250/61.2	OSAS	Shopping	5/high risk of bias	No
Zamboni [9•]	2021/2020	Italy Developed High income 74.39	Online	No	General population	43.25	1196/64.6	One item asking about of control of the behavior	Shopping	1/high risk of bias	Yes

*BSMAS*, Bergen Social Media Addiction Scale; *BPGS*, Brief Problem Gambling Screen; *CGAS-SF*, Children’s Game Addiction Scale-Short Form; *CIUS*, Compulsive Internet Use Scale; *CSBDS*, Compulsive Sexual Behavior Disorder Scale; *EAI*, Exercise Addiction Inventory; *GAS*, Game Addiction Scale; *GAS-A*, Game Addiction Scale for Adolescents; *IAT*, Internet Addiction Test; *IGDS*, Internet Gaming Disorder Scale; *MPAI*, Mobile Phone Addiction Index; *MPPUS*, Mobile Phone Problem Use Scale; *OSAS*, Online Shopping Addiction Scale; *Parent–Child IAT*, Parent–Child Internet Addiction Test; *PG Adaptation of Yale-Brown OCS*, Pathological Gambling Adaptation of Yale-Brown Obsessive Compulsive Scale; *PBS*, Problem Behavior Scale; *PGSI*, Problem Gambling Severity Index; *PRIUSS*, Problematic Internet Use Scales; *SAST*, Sexual Addiction Screening Test; *SAS-SF*, Smartphone Addiction Scale-Short Version; *SABAS*, Smartphone Application-Based Addiction Scale; *SOGS*, South Oaks Gambling Screen; *YFAS*, Yale Food Addiction Scale; *YDQ*, Young’s Diagnostic Questionnaire; *NOS*, Newcastle Ottawa Scale

### Quality Assessment

**Fig. 2:**
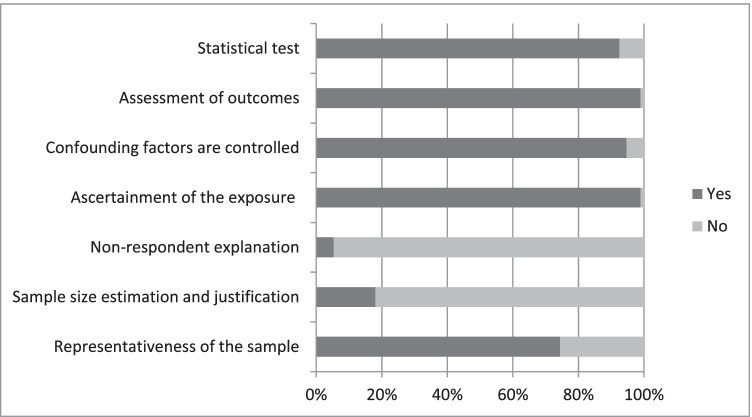
Details of methodological quality assessment based on NOS checklist within included studies

Most of the studies (75 out of 94) were categorized as being high-quality (or low risk of bias) studies. The total score of methodological quality is provided in Table 1 with details in Fig. 2. The main methodological problems were:

i. Most studies (89 out of 94) did not report the description of the response rate or the characteristics of the responders and the non-responders.
ii. Most studies (77 out of 94) did not provide an explanation regarding sample size estimation and justification.
iii. Some studies (44 out of 94) did not recruit a representative sample (i.e., they used a selected group of population or did not provide description regarding the sampling strategy).

### Outcome Measures

#### Pooled Prevalence

**Fig. 3:**
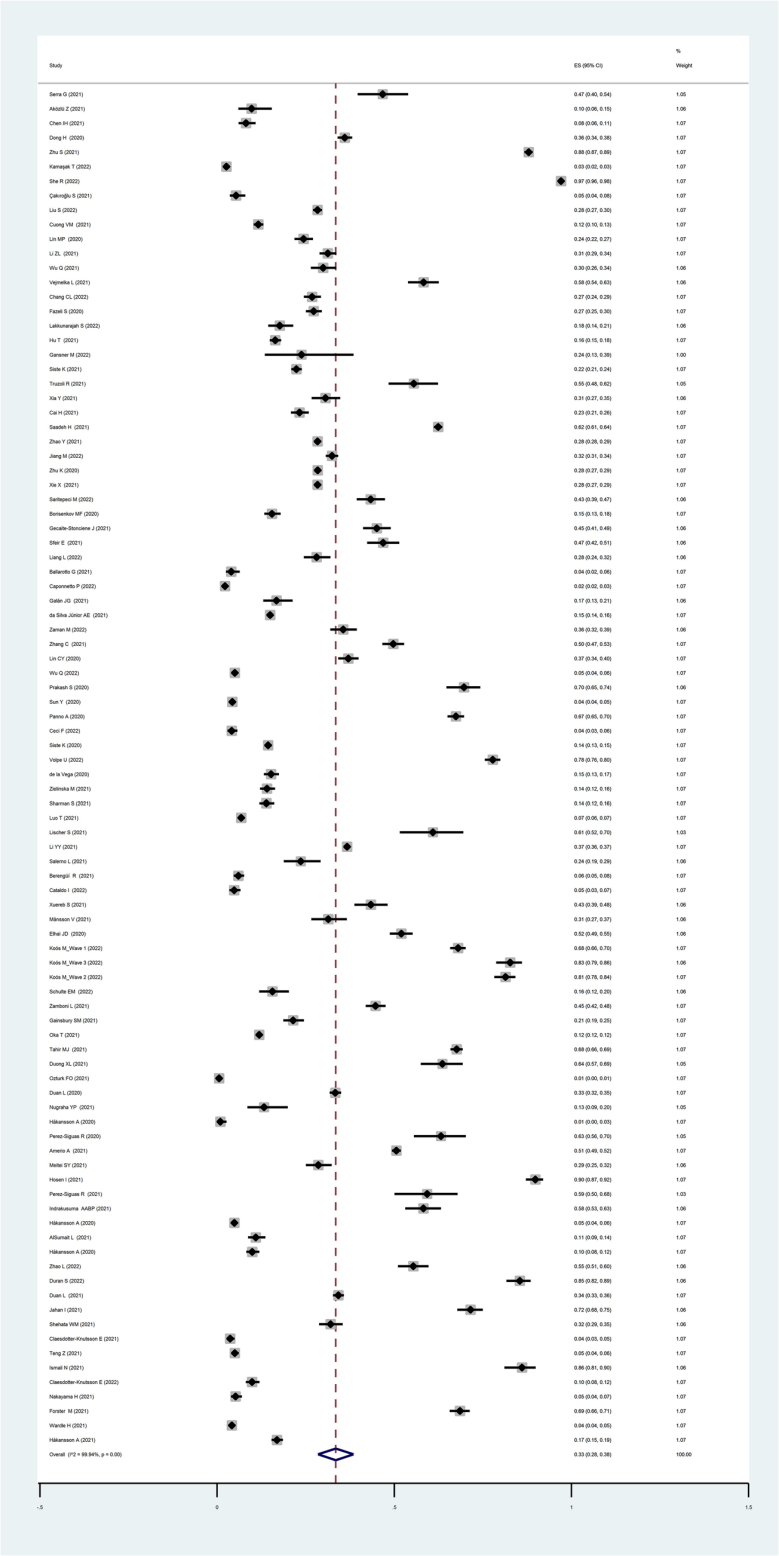
Forest plot regarding the pooled prevalence of all types of behavioral addiction

The pooled estimated prevalence of all types of behavioral addictions was 33% (94 studies, 95% *CI*: 28 to 38%, *I*^2^: 99.94%, τ^2^: 0.06). Figure 3 provides the forest plot regarding the pooled prevalence. The pooled prevalence rates of specific behavioral addictions are listed below:

i. Internet addiction: 30% (39 studies, 95% *CI*: 26 to 34%, *I*^2^: 99.86%, τ.^2^: 0.02)
ii. Gaming addiction: 24% (19 studies, 95% *CI*: 14 to 33%, *I*^2^: 99.92%, τ.^2^: 0.04)
iii. Gambling addiction: 24% (18 studies, 95% *CI*: 17 to 31%, *I*^2^: 99.74%, τ.^2^: 0.02)
iv. Smartphone addiction: 48% (13 studies, 95% *CI*: 36 to 61%, *I*^2^: 99.73%, τ.^2^: 0.05)
v. Social media addiction: 52% (10 studies, 95% *CI*: 30 to 73%, *I*^2^: 99.93%, τ.^2^: 0.12)
vi. Food addiction: 21% (five studies, 95% *CI*: 10 to 32%, *I*^2^: 99.30%, τ.^2^: 0.02)
vii. Sex addiction: 34% (five studies, 95% *CI*: 19 to 49%, *I*^2^: 99.86, τ.^2^: 0.03)
viii. Exercise addiction: 7% (four studies, 95% *CI*: 3 to 12%, *I*^2^: 96.24%, τ.^2^ < 0.001)
ix. Shopping addiction: 10% (two studies, 95% *CI*: 9 to 12%, *I*^2^: not applicable, τ.^2^: not applicable)

#### Publication Bias

**Fig. 4:**
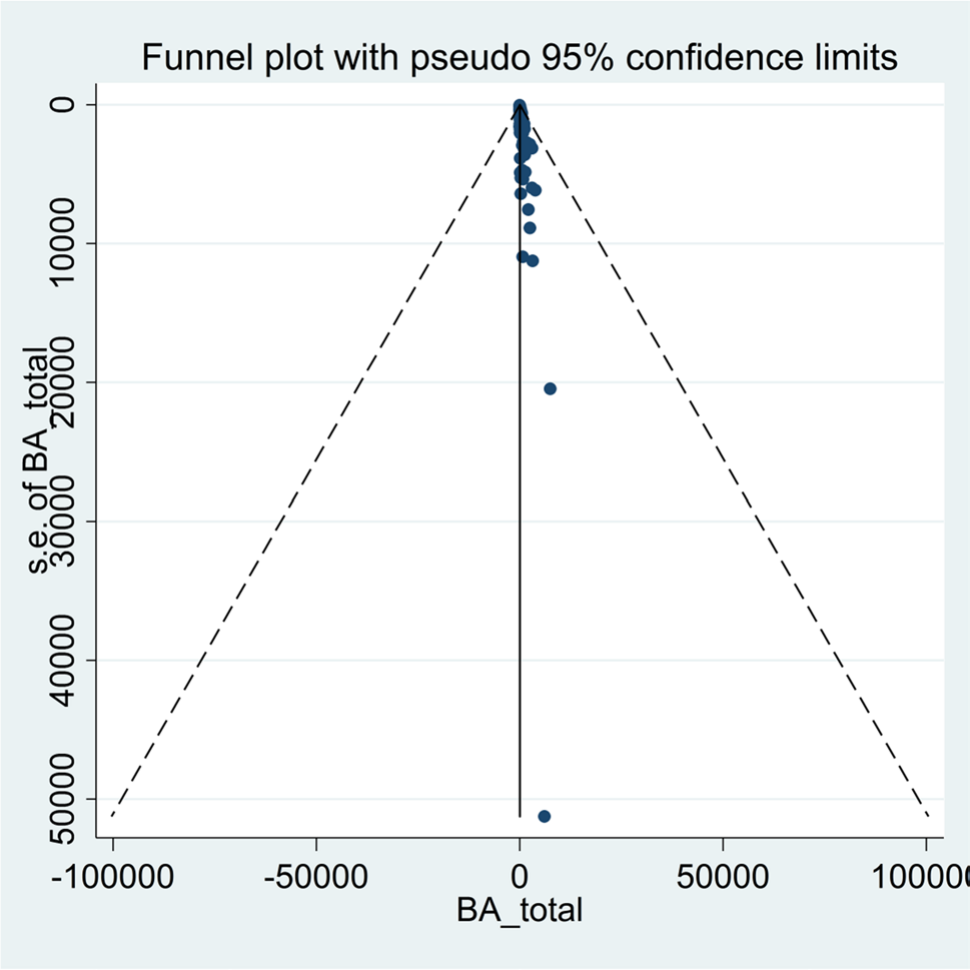
Funnel plot assessing the publication bias among included studies

The probability of publication bias was assessed using Begg’s test (*p* = 0.002) and funnel plot. Based on asymmetric funnel plot (Fig. 4), publication bias seems probable.

#### Correction for Publication Bias

**Fig. 5:**
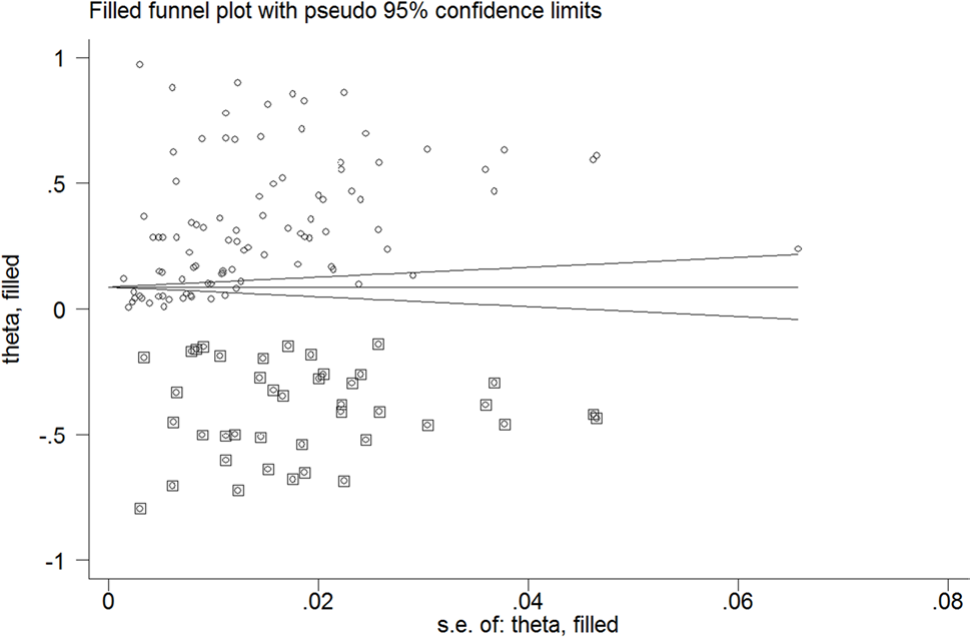
Corrected funnel plot based on the fill and trim method

The fill-and-trim method was used to correct probable publication bias. In this method, 41 studies were imputed, and the corrected pooled prevalence of all types of behavioral addictions was 11.1% (95% *CI*: 5.4 to 16.8%; τ^2^: 0.11; *p* < 0.001). The resultant funnel plot after trimming is provided in Fig. 5. The corrected type specific prevalence rates of behavioral addictions are listed below:

i. Internet addiction: 10.6% (39 studies, 18 imputed studies, 95% *CI*: 6.2 to 15.1%, τ.^2^: 0.03)
ii. Gaming addiction: 5.3% (19 studies, 10 imputed studies, 95% *CI*: 0 to 15.3%, τ.^2^: 0.07)
iii. Gambling addiction: 7.2% (18 studies, 8 imputed studies, 95% *CI*: 0 to 15.4%, τ.^2^: 0.05)
iv. Smartphone addiction: 30.7% (13 studies, six imputed studies, 95% *CI*: 16.3 to 45.2%, τ.^2^: 0.10)
v. Social media addiction: 15.1% (10 studies, five imputed studies, 95% *CI*: 0 to 36.5%, τ.^2^: 0.18)
vi. Sex addiction: 9.4% (five studies, two imputed studies, 95% *CI*: 0 to 24.6%, τ.^2^: 0.04)
vii. Shopping addiction: 7.2% (two studies, one imputed study, 95% *CI*: 0 to 54.3%, τ.^2^: 0.17)

Food addiction and exercise addiction were not affected by publication bias.

#### Sensitivity Analysis

Sensitivity analysis (based on the one-out or Jack-knife method) showed that the pooled effect size was not affected by a single study effect.

#### Moderator Analysis

Moderators of prevalence for all type and specific behavioral addictions were assessed using uni-variable meta-regression (Table 2) and multivariable meta-regression (Table 3).

**Table 2:** Results of uni-variable meta-regression regarding estimated pooled prevalence

Type of behavioral addiction	Variable	Number of studies	Coefficient	S.E	*p*	*I*^2^ res. (%)	Adj. *R*^2^ (%)	τ^2^
All types	Country developmental status (developed vs. developing)	94	− 0.06	0.05	0.27	99.93	0.25	0.07
	Country income level (high, upper-middle, lower-middle)	94	− 0.05	0.04	0.14	99.94	1.33	0.06
	Individuals using the Internet (% of population)	91	− 0.003	0.001	0.03	99.93	4.13	0.06
	Data collection method (online vs. others)	94	0.03	0.07	0.69	99.91	− 0.92	0.07
	Lockdown period (yes vs. no)	94	0.04	0.06	0.47	99.94	− 0.52	0.07
	Population based vs. selected groups	94	− 0.02	0.06	0.73	99.94	− 0.96	0.07
	Participant groups	94	− 0.02	0.01	0.24	99.94	0.46	0.06
	Mean age of participants	66	0.002	0.003	0.53	99.94	− 0.94	0.06
	Female percentage of participants	90	0.002	0.002	0.33	99.94	− 0.03	0.06
	Methodological quality (low vs. high risk of bias)	94	0.04	0.07	0.51	99.94	− 0.61	0.07
Internet addiction	Country developmental status (developed vs. developing)	39	− 0.06	0.07	0.39	99.79	− 0.58	0.04
	Individuals using the Internet (% of population)	38	− 0.002	0.002	0.39	99.81	− 0.60	0.04
	Country income level (high, upper-middle, lower-middle)	39	− 0.04	0.05	0.37	99.86	− 0.42	0.04
	Data collection method (online vs. others)	39	0.08	0.08	0.32	99.83	0.03	0.04
	Lockdown period (yes vs. no)	39	0.06	0.08	0.47	99.86	− 1.25	0.04
	Population based vs. selected groups	39	0.13	0.10	0.20	99.86	1.84	0.04
	Participant groups	39	0.007	0.02	0.71	99.86	− 2.32	0.05
	Mean age of participants	28	− 0.0001	0.003	0.96	99.85	− 3.93	0.03
	Female percentage of participants	36	0.001	0.003	0.77	99.87	− 2.69	0.05
	Methodological quality (low vs. high risk of bias)	39	0.08	0.09	0.39	99.86	− 0.46	0.04
Gaming addiction	Country developmental status (developed vs. developing)	19	0.05	0.10	0.62	99.92	− 4.30	0.05
	Country income level (high, upper-middle, lower-middle)	19	− 0.01	0.07	0.87	99.91	− 5.70	0.05
	Individuals using the Internet (% of population)	19	− 0.003	0.003	0.23	99.91	3.08	0.04
	Data collection method (online vs. others)	19	− 0.20	0.11	0.09	99.83	11.33	0.04
	Lock down period (yes vs. no)	19	0.18	0.09	0.06	99.88	14.52	0.04
	Population based vs. selected groups	19	0.02	0.11	0.82	99.93	− 5.56	0.05
	Participant groups	19	0.04	0.04	0.27	99.90	1.67	0.04
	Mean age of participants	15	− 0.004	0.005	0.44	99.90	− 2.64	0.05
	Female percentage of participants	19	− 0.001	0.005	0.79	99.92	− 5.40	0.05
	Methodological quality (low vs. high risk of bias)	19	− 0.006	0.14	0.97	99.92	− 5.83	
Gambling addiction	Country developmental status (developed vs. developing)	All were conducted in developed countries with high income level						
	Country income level (high, upper-middle, lower-middle)							
	Individuals using the Internet (% of population)	18	− 0.02	0.005	0.005	98.36	38.56	0.02
	Data collection method (online vs. others)	All studies collected data via online method						
	Lock down period (yes vs. no)	18	0.08	0.09	0.38	99.75	− 0.88	0.03
	Population based vs. selected groups	18	− 0.004	0.09	0.96	99.72	− 6.41	0.03
	Participant groups	18	− 0.001	0.02	0.97	99.72	− 6.38	0.03
	Mean age of participants	10	− 0.0001	0.01	0.99	96.92	− 13.73	0.02
	Female percentage of participants	18	0.0003	0.003	0.90	99.71	− 6.21	0.03
	Methodological quality (low vs. high risk of bias)	18	− 0.23	0.12	0.09	99.22	12.24	0.03
Smartphone addiction	Country developmental status (developed vs. developing)	13	− 0.15	0.15	0.34	99.74	− 0.06	0.04
	Country income level (high, upper-middle, lower-middle)	13	− 0.195	0.08	0.04	99.65	26.93	0.03
	Individuals using the Internet (% of population)	13	− 0.006	0.003	0.05	99.73	23.93	0.03
	Data collection method (online vs. others)	All studies collected data via online method						
	Lockdown period (yes vs. no)	13	− 0.01	0.21	0.95	99.75	− 9.02	0.04
	Population based vs. selected groups	None of the studies were population based						
	Participant groups	13	− 0.01	0.04	0.78	99.69	− 8.26	0.04
	Mean age of participants	6	0.003	0.006	0.61	99.79	− 15.61	0.03
	Female percentage of participants	13	− 0.001	0.004	0.79	99.75	− 8.35	0.04
	Methodological quality (low vs. high risk of bias)	13	− 0.45	0.16	0.02	99.62	36.33	0.02
Social media addiction	Country developmental status (developed vs. developing)	10	0.27	0.22	0.24	99.91	6.21	0.10
	Country income level (high, upper-middle, lower-middle)	10	0.09	0.16	0.61	99.93	− 8.62	0.11
	Individuals using the Internet (% of population)	10	0.02	0.10	0.16	99.89	13.55	0.09
	Data collection method (online vs. others)	10	− 0.02	0.36	0.96	99.93	− 12.48	0.12
	Lockdown period (yes vs. no)	10	0.29	0.19	0.17	99.88	12.29	0.09
	Population based vs. selected groups	10	0.15	0.22	0.52	99.93	− 6.52	0.11
	Participant groups	10	− 0.02	0.07	0.79	99.94	− 11.41	0.12
	Mean age of participants	9	0.02	0.01	0.15	99.94	16.35	0.09
	Female percentage of participants	9	− 0.02	0.01	0.05	99.94	36.77	0.06
	Methodological quality (low vs. high risk of bias)	10	0.39	0.23	0.13	99.94	16.90	0.09
Food addiction	Country developmental status (developed vs. developing)	5	0.07	0.18	0.71	99.47	− 26.37	0.03
	Country income level (high, upper-middle, lower-middle)	5	0.10	0.14	0.51	99.27	− 12.20	0.02
	Individuals using the Internet (% of population)	5	− 0.008	0.01	0.48	99.47	− 10.25	0.02
	Data collection method (online vs. others)	All studies collected data via online method						
	Lock down period (yes vs. no)	5	0.32	0.01	< 0.001	0	100	< 0.001
	Population based vs. selected groups	5	0.10	0.14	0.51	99.27	− 12.20	0.02
	Participant groups	5	0.10	0.14	0.51	99.27	− 12.20	0.02
	Mean age of participants	5	− 0.002	0.10	0.88	99.46	− 32.13	0.03
	Female percentage of participants	5	− 0.0001	0.006	0.99	99.47	− 33.35	0.03
	Methodological quality (low vs. high risk of bias)	5	0.07	0.18	0.72	99.47	− 26.51	0.03
Sex addiction	Country developmental status (developed vs. developing)	All were population-based studies conducted in developed countries with high income level using online data collection method						
	Country income level (high, upper-middle, lower-middle)							
	Data collection method (online vs. others)							
	Population based vs. selected groups							
	Lock down period (yes vs. no)	5	0.41	0.31	0.27	99.88	16.96	0.08
	Individuals using the Internet (% of population)	5	0.09	0.008	0.002	93.15	96.90	0.003
	Mean age of participants	5	0.02	0.02	0.35	99.90	5.64	0.09
	Female percentage of participants	5	− 0.03	0.02	0.16	99.87	38.36	0.06
	Methodological quality (low vs. high risk of bias)	5	0.41	0.31	0.27	99.88	16.96	0.08

N.B. exercise (four studies) and shopping (two studies) did not have sufficient data for moderator analysis. *S.E*, standard error; *I*^*2*^* res*, *I*^2^ residual; *Adj R*^*2*^, adjusted *R*^2^

**Table 3:** Results of multivariable meta-regression regarding estimated pooled prevalence

Type of behavioral addiction	Variable	Number of studies	Coefficient	S.E	*p*	*I*^2^ res. (%)	Adj. *R*^2^ (%)	τ^2^
All types	Individuals using the Internet (% of population)	91	− 0.003	0.001	0.05	99.93	4.23	0.06
	Participants group		− 0.01	0.01	0.30			
Gaming addiction	Lockdown period (yes vs. no)	19	0.21	0.09	0.03	99.77	31.10	0.03
	Individuals using the Internet (% of population)		− 0.001	0.003	0.57			
	Data collection method (online vs. others)		− 0.24	0.10	0.04			
	Participants group		0.01	0.04	0.74			
Gambling addiction	Individuals using the Internet (% of population)	18	− 0.02	0.01	0.03	98.45	34.28	0.02
	Methodological quality (low vs. high risk of bias)		0.04	0.16	0.82			
Smartphone addiction	Country income level (high, upper-middle, lower-middle)	13	− 0.17	0.14	0.27	99.35	34.53	0.03
	Individuals using the Internet (% of population)		0.003	0.006	0.61			
	Methodological quality (low vs. high risk of bias)		− 0.41	0.24	0.12			
Social media addiction	Female percentage of participants	9	− 0.05	0.008	0.03	97.30	93.67	0.006
	Mean age of participants		− 0.01	0.006	0.19			
	Lockdown period (yes vs. no)		2.08	0.57	0.06			
	Individuals using the Internet (% of population)		− 0.08	0.03	0.13			
	Country developmental status (developed vs. developing)		0.52	0.27	0.19			
	Methodological quality (low vs. high risk of bias)		− 1.57	0.57	0.10			

##### All Types of Behavioral Addiction

Based on uni-variable meta-regression, the percentage of individuals using the internet in the country was the only significant moderator in all types of behavioral addictions, accounting for 4.23% of variance. Each percentage increase of individuals using the internet in the country was associated with 0.3% decrease in all types of behavioral addiction prevalence rates. Other examined variables did not affect pooled prevalence or heterogeneity.

##### Internet Addiction

Based on uni-variable meta-regression, none of the examined variables affect pooled prevalence or heterogeneity of internet addiction.

##### Gaming Addiction

Based on multivariable meta-regression, data collection method (online vs. other methods, *p* = 0.04) and lockdown period (yes vs. no, *p* = 0.03) were significant predictors of gaming addiction during the COVID-19 pandemic. The prevalence rate of gaming addiction was 24% lower in studies with online data collection method vs. studies using other data collection methods. The prevalence rate of gaming addiction was 21% higher during lockdown period vs. non-lockdown period. These variables explained 31.01% variance in the prevalence of gaming addiction.

##### Gambling Addiction

Based on multivariable meta-regression, the percentage of individuals using the internet in the country was the only significant moderator in gambling prevalence (*p* = 0.03), accounting for 34.28% of variance in prevalence of gambling. Each 1% increase of individuals using the internet in each country was associated with a 1.6% decrease in gambling prevalence.

##### Smartphone Addiction

Based on uni-variable meta-regression, country income level (high, upper-middle, lower-middle, *p* = 0.04), percentage of individuals using the internet in the country (*p* = 0.05), and methodological quality (low vs. high risk of bias, *p* = 0.02) were moderators of smartphone addiction. Based on multivariable meta-regression models, the prevalence of smartphone addiction in low risk of bias studies was 41% lower than in high risk of bias studies. The prevalence rate of smartphone addiction was 27% (95% *CI*: 24 to 29%) in high-income countries, 45% (95% *CI*: 32 to 58%) in upper intermediate income countries, and 84% (95% *CI*: 82 to 86%) in lower intermediate income countries. Each 1% increase of individuals using the internet in the country was associated with a 0.3% decrease in smartphone addiction prevalence. These variables accounted for 34.53% of variance in the prevalence of smartphone addiction.

##### Social Media Addiction

Based on multivariable meta-regression, the female percentage of participants (each 1% increase in female participants was associated with a 4.6% decrease in social media addiction, *p* = 0.03); being in lockdown period (two times higher than in non-lockdown period, *p* = 0.06); mean age of participants (each year increase was associated with 1.1% decrease in social media addiction, *p* = 0.19); percentage of individuals using the internet in country (each 1% increase of individuals using the internet in the country was associated with an 8.3% decrease in social media addiction prevalence, *p* = 0.13); developing status of country (52.5% higher in developed vs. developing countries, *p* = 0.19); and methodological quality of studies (1.5 times lower in low risk of bias vs. high risk of bias studies, *p* = 0.10) were predictors of social media addiction, accounting for 93.67% of the variance.

##### Food Addiction

Based on uni-variable meta-regression, being in lockdown period (yes vs. no, *p* < 0.001) was the only significant predictor of food addiction which accounted for 100% of the variance. The prevalence rate of food addiction was 32% higher during the lockdown period vs. non-lockdown period.

##### Sex Addiction

Based on uni-variable meta-regression, the percentage of individuals using the internet in the country (*p* = 0.002) was the only significant predictor of sex addiction which accounted for 96.90% of the variance. Each 1% increase of individuals using the internet in the country was associated with a 9% increase in sex addiction prevalence.

Exercise addiction (four studies) and shopping addiction (two studies) did not have sufficient data for moderator analysis.

## Discussion

Due to the COVID-19 pandemic, human behaviors have changed substantially [149]. Therefore, it is important for healthcare providers and government authorities to understand the changed behaviors, especially addictive behaviors, during the COVID-19 pandemic. Therefore, healthcare providers and government authorities could consider appropriate programs to respond to behavioral addiction issues. The present systematic review and meta-analysis therefore used a rigorous methodology to estimate the prevalence of overall behavioral addictions (comprising internet addiction, smartphone addiction, gaming addiction, social media addiction, food addiction, exercise addiction, gambling addiction, and shopping addiction) during the COVID-19 pandemic and associated factors using meta-regression. Moreover, the prevalence rate of each individual behavioral addiction was reported and tested for its associated factors.

The findings showed that the corrected pooled prevalence of overall behavioral addictions was 11.1% (95% *CI*: 5.4% to 16.8%), and the corrected prevalence rates of each behavioral addiction varied between 7% (exercise addiction) and 30.7% (smartphone addiction). Moreover, the female percentage of participants, mean age of participants, percentage of individuals using the internet in the country, and the developing status of the country were moderators of social media addiction prevalence. Methodological quality of studies was associated with social media addiction and smartphone addiction prevalence. Being in lockdown period was a moderator of the prevalence rates for food addiction, gaming addiction, and social media addiction. Individuals using the internet (percentage of the population) were associated with overall prevalence rates for behavioral addiction, sex addiction, and gambling addiction. Data collection method (online vs. other methods) was associated with the prevalence of gaming addiction.

Before the COVID-19 pandemic, addictive behaviors had been identified as an important factor affecting individuals’ health, such as sleep quality and quality of life [150–159]. Among the different types of addictive behaviors, internet addiction has been studied with growing interest because of technology advancement [160]. Moreover, the internet has been considered as a medium for individuals to engage in different activities. With the convenience of internet use, especially the technology advancement in smartphones (i.e., smartphones are user-friendly with internet access and power apps functions), individuals are likely to become addicted to different types of activities (e.g., social media use, online shopping, and online gaming). Smartphone use is similar to internet use because it provides another medium for individuals to easily engage in different activities and provides the potential for smartphone addiction [161]. Therefore, the high prevalence rates of internet addiction (10.6%) and smartphone addiction (30.7%) found in the present systematic review and meta-analysis are likely explained by the nature of being a 24/7 medium.

In contrast, prevalence rates of shopping addiction (7.2%) and exercise addiction (7.0%) were not high (relatively) in the present study’s findings. The main reason could be the countries’ policies in COVID-19 infection control. More specifically, governments encouraged citizens and residents to reduce outdoor activities and many closed facilities for commercial or exercise purposes (e.g., mall and gym closure) [14–16]. Therefore, individuals who had a problem of shopping addiction or exercise addiction were somewhat restricted in their addictive behaviors (i.e., shopping and exercise). However, some are likely to have adapted their addictive behaviors to satisfy their cravings (e.g., physical shopping changing to online shopping; exercise in a gym changing to home exercise); the changed environments might somewhat decrease their desire in engaging in such addictive behaviors.

The present systematic review and meta-analysis further identified that the lockdown period was a significant factor associated with prevalence of several behavioral addictions (including food addiction, gaming addiction, and social media addiction). The finding that lockdown period had higher prevalence rate of overall behavioral addiction than non-lockdown period could be explained by the internet advancement and individuals’ coping strategies during the lockdown period. More specifically, lockdown may have increased individuals’ psychological distress and individuals may have engaged in some potentially addictive behaviors to cope with their psychological distress. Therefore, some individuals are likely to develop behavioral addictions to cope with their psychological distress, and this mechanism echoes the I-PACE model proposed by Brand et al. [17].

Individuals using the internet (as a percentage of the population) were found to be another significant factor contributing to the behavioral addictions. This finding could be explained by the peer effect [162]. More specifically, when individuals found that their friends and family members were all constantly using the internet, they may have felt that using internet constantly was socially acceptable. Such a feeling may motivate those who have behavioral addictions via an internet platform to keep engaging in their online behavioral addictions. As a result, when the country has a higher percentage of individuals using the internet, the society is likely to have a higher rate of prevalence in behavioral addictions.

Based on the findings of the present systematic review and meta-analysis, there are several implications. First, if a lockdown is needed to control infection and disease, healthcare providers and government authorities should pay special attention to the possibility of increased behavioral addictions among their citizens. Different programs such as online cognitive behavioral therapy and online mindfulness programs may be provided to help individuals go through the tough lockdown period without increasing their craving for their addictive behavior of choice. Second, governments should be alerted when they observe a high percentage of individuals using internet. Appropriate programs or policies may be designed for those countries with a high percentage of individuals using the internet to prevent consequent behavioral addiction problems.

### Limitations

The present study has a number of limitations. First, some of the analyzed studies did not have representative samples. Therefore, the estimated prevalence reported in the present systematic review and meta-analysis might not have good generalizability to the entire population worldwide. Additionally, the response rates were unclear for most of the analyzed studies. Therefore, the representativeness of the studied samples is arguably problematic. Second, most of the studies used online surveys to collect the data, which may cause selection bias in sampling. More specifically, individuals without internet access or those who did not use internet during the survey period were unable to complete the survey assessing their behavioral addictions. Therefore, the estimations on internet-related addictive behaviors could be overestimated (because those who did not use internet were not included in the present study). Third, almost all the studies analyzed in the present systematic review and meta-analysis used a cross-sectional design, which lacks the ability to determine causal relationships between the study variables. Lastly, the information was imbalanced between different types of behavioral addictions (e.g., most studies reported for addictions to internet use and smartphone use, and only two studies reported addictions to shopping). Therefore, the prevalence rates of the behavioral addictions reported from few studies have the issue of small sample sizes and probable low heterogeneity.

## Conclusion

Behavioral addictions are potential health issues during the COVID-19 pandemic. High prevalence rates of different types of behavioral addictions have been estimated with the use of a rigorous methodology in the present meta-analysis. Given that behavioral addictions are associated with a variety of health issues and subsequently cause care burden for the societies, healthcare providers and government authorities should pay attention to the issue of behavioral addictions during the COVID-19 pandemic. Indeed, several statements have been announced for government authorities and related stakeholders to take care of the issues of behavioral addictions [47, 49, 163]. The findings in the present systematic review and meta-analysis echo the importance of these statements. Therefore, designing appropriate programs to reduce behavioral addictions during the COVID-19 pandemic (and for subsequent pandemics) is highly recommended.

## Supplementary Information

Below is the link to the electronic supplementary material.

Supplementary file1 (DOCX 30 kb)

## Data Availability

All tables and figures are original and have been produced by the authors for this publication. Tables and figures have not previously been published.
