# Supplementary material for: Estimation of Behavioral Addiction Prevalence During COVID-19 Pandemic: A Systematic Review and Meta-analysis
Source: Curr Addict Rep. 2022 Sep 12;9(4):486–517. doi: 10.1007/s40429-022-00435-6 (PMC9465150; doi:10.1007/s40429-022-00435-6)
Supplement: Supplementary file 1 — Supplementary file1 (DOCX 30 kb) [file 40429_2022_435_MOESM1_ESM.docx]

**Supplement 1: Search syntax adapted for each database**

**Title: Estimation of behavioral addiction prevalence during COVID-19 pandemic: A systematic review and meta-analysis**

***PubMed***

(internet[tiab] OR “social media”[tiab] OR “smartphone”[tiab] OR “mobile phone”[tiab] OR “cell phone”[tiab] OR gaming[tiab] OR (video[tiab] AND gam*[tiab]) OR (social[tiab] AND network*[tiab]) OR Twitter[tiab] OR Instagram[tiab] OR “YouTube”[tiab] OR “Facebook”[tiab] OR “WhatsApp”[tiab] OR “TikTok”[tiab] OR “WeChat”[tiab] OR “SnapChat”[tiab] OR “QQ”[tiab] OR “Tinder”[tiab] OR gambl*[tiab] OR betting[tiab] OR “electronic gaming machines”[tiab] OR lotto[tiab] OR casino[tiab] OR poker[tiab] OR bingo[tiab] OR blackjack[tiab] OR lottery[tiab] OR (slot[tiab] AND machine*[tiab]) OR exercis*[tiab] OR “physical activity”[tiab] OR pornography[tiab] OR sex*[tiab] OR “binge watching”[tiab] OR viewing[tiab] OR food[tiab] OR “binge eating”[tiab] OR mukbang[tiab] OR shopping[tiab] OR buying[tiab] OR technolog*[tiab] OR “work addiction”[tiab]) AND (addict*[tiab] OR problem*[tiab] OR depend*[tiab] OR disorder*[tiab] OR obsess*[tiab] OR excess*[tiab] OR compuls*[tiab] OR impuls*[tiab] OR excess*[tiab]) AND (“SARS-CoV-2”[tiab] OR “coronavirus”[tiab] OR “COVID-19”[tiab] OR “2019-nCoV”[tiab] OR “coronavirus disease-2019”[tiab] OR covid[tiab] OR “2019-ncov”[tiab] OR “sars-cov-2”[tiab] OR “cov-19”[tiab])

***Scopus***

(TITLE-ABS (internet) OR TITLE-ABS (“social media”) OR TITLE-ABS (smartphone) OR TITLE-ABS (“mobile phone”) OR TITLE-ABS(“cell phone”) OR TITLE-ABS(gaming) OR TITLE-ABS (“video gam*”) OR TITLE-ABS (“social network*”) OR TITLE-ABS(Twitter) OR TITLE-ABS(Instagram) OR TITLE-ABS(“YouTube”) OR TITLE-ABS (“Facebook”) OR TITLE-ABS (“WhatsApp”) OR TITLE-ABS(“TikTok”) OR TITLE-ABS(“WeChat”) OR TITLE-ABS(“SnapChat”) OR TITLE-ABS (“QQ”) OR TITLE-ABS (“Tinder”) OR TITLE-ABS (gambl*) OR TITLE-ABS (betting) OR TITLE-ABS (“electronic gaming machines”) OR TITLE-ABS (lotto) OR TITLE-ABS (casino) OR TITLE-ABS (poker) OR TITLE-ABS (bingo) OR TITLE-ABS (blackjack) OR TITLE-ABS (lottery) OR TITLE-ABS (“slot machine*”) OR TITLE-ABS (exercis*) OR TITLE-ABS (“physical activity”) OR TITLE-ABS (pornography) OR TITLE-ABS (sex*) OR TITLE-ABS (“binge watching”) OR TITLE-ABS(viewing) OR TITLE-ABS(food) OR TITLE-ABS (“binge eating”) OR TITLE-ABS(mukbang) OR TITLE-ABS (shopping) OR TITLE-ABS (buying) OR TITLE-ABS (technolog*)) AND (TITLE-ABS(addict*) OR TITLE-ABS (problem*) OR TITLE-ABS (depend*) OR TITLE-ABS (disorder*) OR TITLE-ABS (obsess*) OR TITLE-ABS (excess*) OR TITLE-ABS (compuls*) OR TITLE-ABS (impuls*) OR TITLE-ABS (excess*)) AND (TITLE-ABS (“SARS-CoV-2”) OR TITLE-ABS (“coronavirus”) OR TITLE-ABS (“COVID-19”) OR TITLE-ABS (“2019-nCoV”) OR TITLE-ABS (“coronavirus disease-2019”) OR TITLE-ABS (covid) OR TITLE-ABS (coronavirus) OR TITLE-ABS (“2019-ncov”) OR TITLE-ABS (“sars-cov-2”) OR TITLE-ABS (“cov-19”))

***ISI Web of Knowledge***

(TS=(internet) OR TS=(“social media”) OR TS=(smartphone) OR TS=(“mobile phone”) OR TS=(“cell phone”) OR TS=(gaming) OR TS=(“video gam*”) OR TS=(“social network*”) OR TS=(Twitter) OR TS=(Instagram) OR TS=(“YouTube”) OR TS=(“Facebook”) OR TS=(“WhatsApp”) OR TS=(“TikTok”) OR TS=(“WeChat”) OR TS=(“SnapChat”) OR TS=(“QQ”) OR TS=(“Tinder”) OR TS=(gambl*) OR TS=(betting) OR TS=(“electronic gaming machines”) OR TS=(lotto) OR TS=(casino) OR TS=(poker) OR TS=(bingo) OR TS=(blackjack) OR TS=(lottery) OR TS=(slot machine*) OR TS=(exercis*) OR TS=(“physical activity”) OR TS=(pornography) OR TS=(sex*) OR TS=(“binge watching”) OR TS=(viewing) OR TS=(food) OR TS=(“binge eating”) OR TS=(mukbang) OR TS=(shopping) OR TS=(buying) OR TS=(technolog*)) AND (TS=(addict*) OR TS=(problem*) OR TS=(depend*) OR TS=(disorder*) OR TS=(obsess*) OR TS=(excess*) OR TS=(compuls*) OR TS=(impuls*) OR TS=(excess*)) AND (TS=(“SARS-CoV-2”) OR TS=(“coronavirus”) OR TS=(“COVID-19”) OR TS=(“2019-nCoV”) OR TS=(“coronavirus disease-2019”) OR TS=(“2019-ncov”) OR TS=(“sars-cov-2”) OR TS=(“cov-19”) OR TS=(COVID 19))

**ProQuest**

(internet OR “social media” OR smartphone OR “mobile phone” OR “cell phone” OR gaming OR “video gam*” OR “social network*” OR Twitter OR Instagram OR “YouTube” OR “Facebook” OR “WhatsApp” OR “TikTok” OR “WeChat” OR “SnapChat” OR “QQ” OR “Tinder” OR gambl* OR betting OR “electronic gaming machines” OR lotto OR casino OR poker OR bingo OR blackjack OR lottery OR “slot machine*” OR exercis* OR “physical activity” OR pornography OR sex* OR “binge watching” OR viewing OR food OR “binge eating” OR mukbang OR shopping OR buying OR technolog*) AND (addict* OR problem* OR depend* OR disorder* OR obsess* OR excess* OR compuls* OR impuls* OR excess*) AND (“SARS-CoV-2” OR “coronavirus” OR “COVID-19” OR “2019-nCoV” OR “coronavirus disease-2019” OR covid OR coronavirus OR “2019-ncov” OR “sars-cov-2” OR “cov-19”)
